# Supplementary material for: Distinct gene-selective roles for a network of core promoter factors in Drosophila neural stem cell identity
Source: Biol Open. 2019 Apr 15;8(4):bio042168. doi: 10.1242/bio.042168 (PMC6504003; doi:10.1242/bio.042168)
Supplement: Supplementary information [file biolopen-8-042168-s1.pdf]

Figure S1

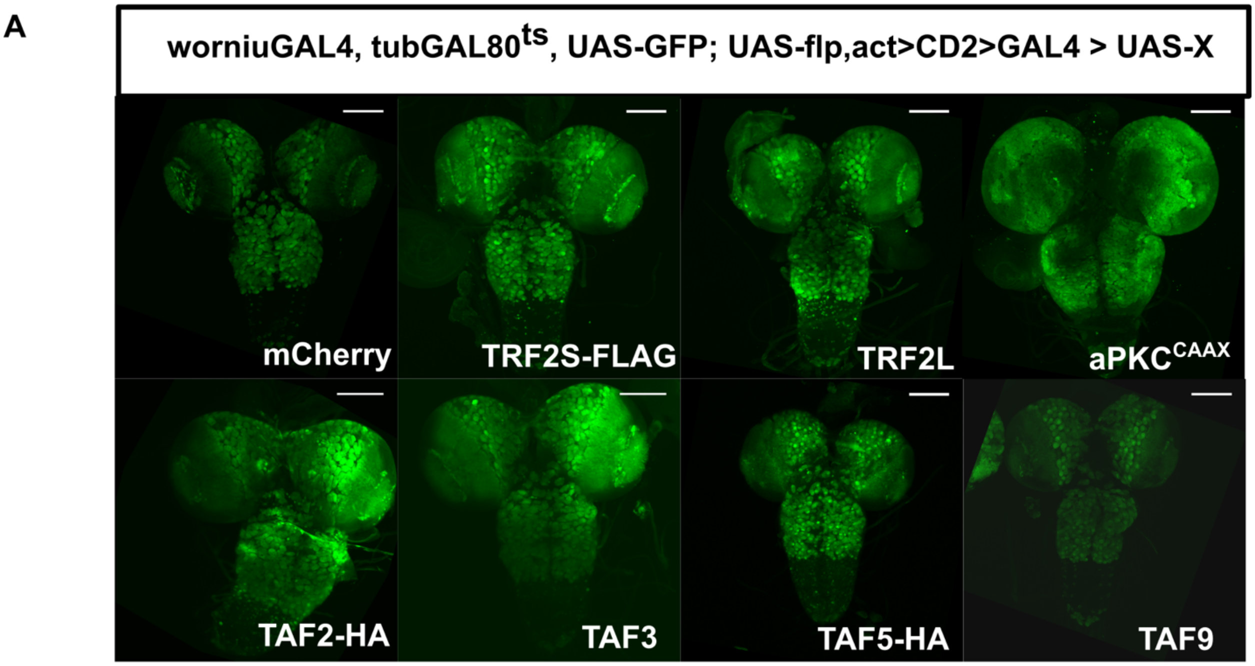

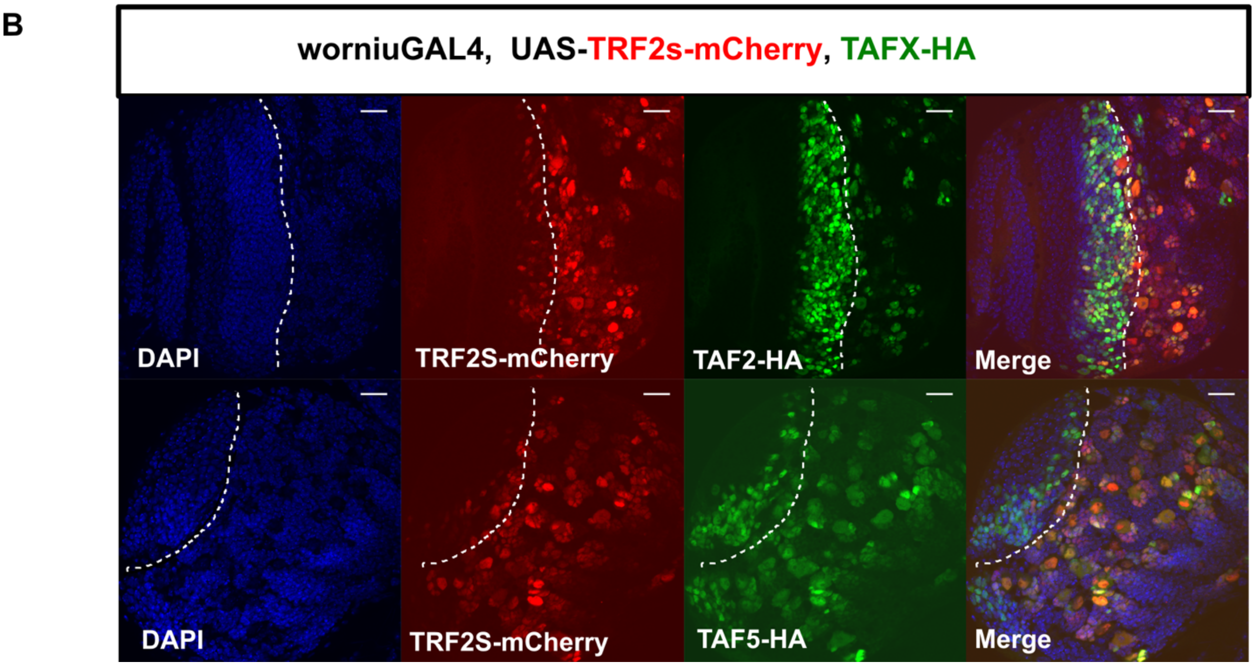

Biology Open • Supplementary information

Figure S1. Overexpression of single TAFs or TRF2 does not result in overt phenotypes. (A) Listed transgenes were overexpressed with a worniu-GAL4, tubGAL80^{ts}, UASGFP; UAS-flp, act>CD2>GAL4 driver. UAS-aPKC^{CAAX} was used as a positive control for increased NSCs. (B) Overexpression of TRF2-mCherry, TAF2-HA, TAF5-HA were confirmed with staining for mCherry (red) and HA (green). The white dotted line demarcates the optic lobe (left)/ central brain (right) boundary.

Scale bars represent 50 μm in A and 20 μm in B.

Figure S2

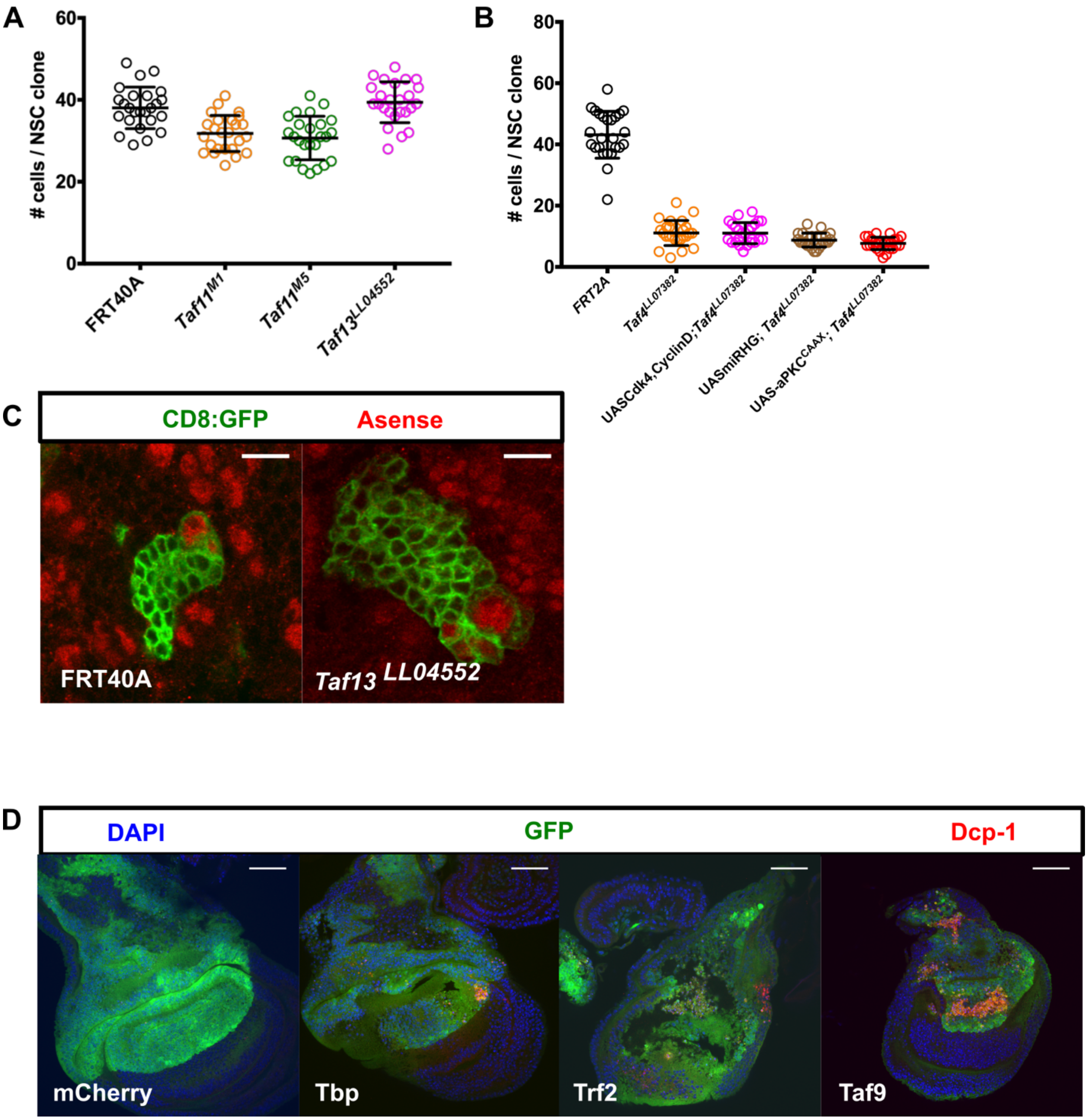

Biology Open • Supplementary information

Figure S2. Analysis of alleles of non-NSC-TAFs and survival function for NSC-TAFs in epithelial cells. (A) $Taf11^{M1}$ and $Taf11^{M5}$ clones exhibit a very modest decrease in clone size (p value <0.0001) whereas $Taf13^{LL04552}$ clones were indistinguishable from control clones (p value = 0.6465). (B) Overexpression of CyclinD, Cdk4,UASmiRHG or UAS-aPKC^{CAAX} does not rescue the small clone size of $Taf4^{LL07382}$ mutant NSCs. Clones were compared to either the control (FRT2A) or the $Taf4^{LL07382}$ mutant clones using a one-way ANOVA with Dunnett's multiple comparison test. (C) $Taf13^{LL04552}$ NSCs express normal levels of the type I NSC marker Asense. Control (FRT40A) or mutant ($Taf13^{LL04552}$) clones are marked with a membrane-tethered GFP, CD8:GFP. (D) Expression of GFP (green) driven by apterous-GAL4, tubGAL80^{ts} and cleaved caspase (red; Dcp-1) in wing discs expressing listed RNAi transgenes induced for 48h, targeting mCherry (control), TBP, TRF2 and TAF9. Scale bars represent 10μm in C and 50 μm in D.

Figure S3

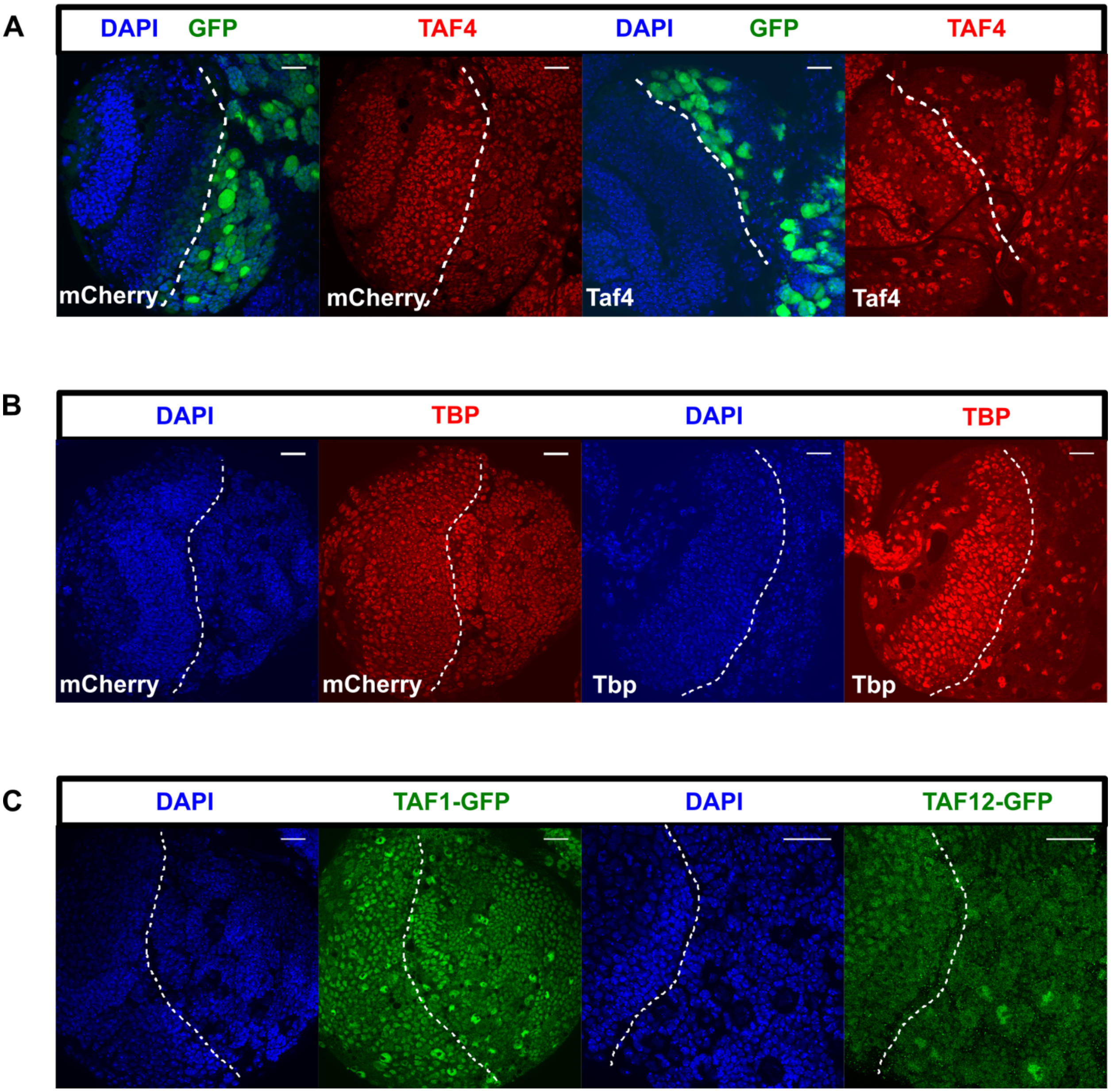

Biology Open • Supplementary information

Figure S3. Endogenous expression of selected TAFs and TBP.

(A). Endogenous TAF4 is expressed ubiquitously in the central brain and expression of a TAF4 RNAi transgene markedly decreases TAF4 levels in GFP-expressing cells. (B) Endogenous TBP is expressed ubiquitously in the central brain and expression of a TBP RNAi transgene markedly decreases TBP levels. (C) TAF1 and TAF12 are expressed ubiquitously in the central brain as reported by TAF1-GFP and TAF12-GFP fusion proteins. The white dotted line demarcates the optic lobe (left)/ central brain (right) boundary and scale bars represent 20 μm.

Biology Open • Supplementary information

Figure S4

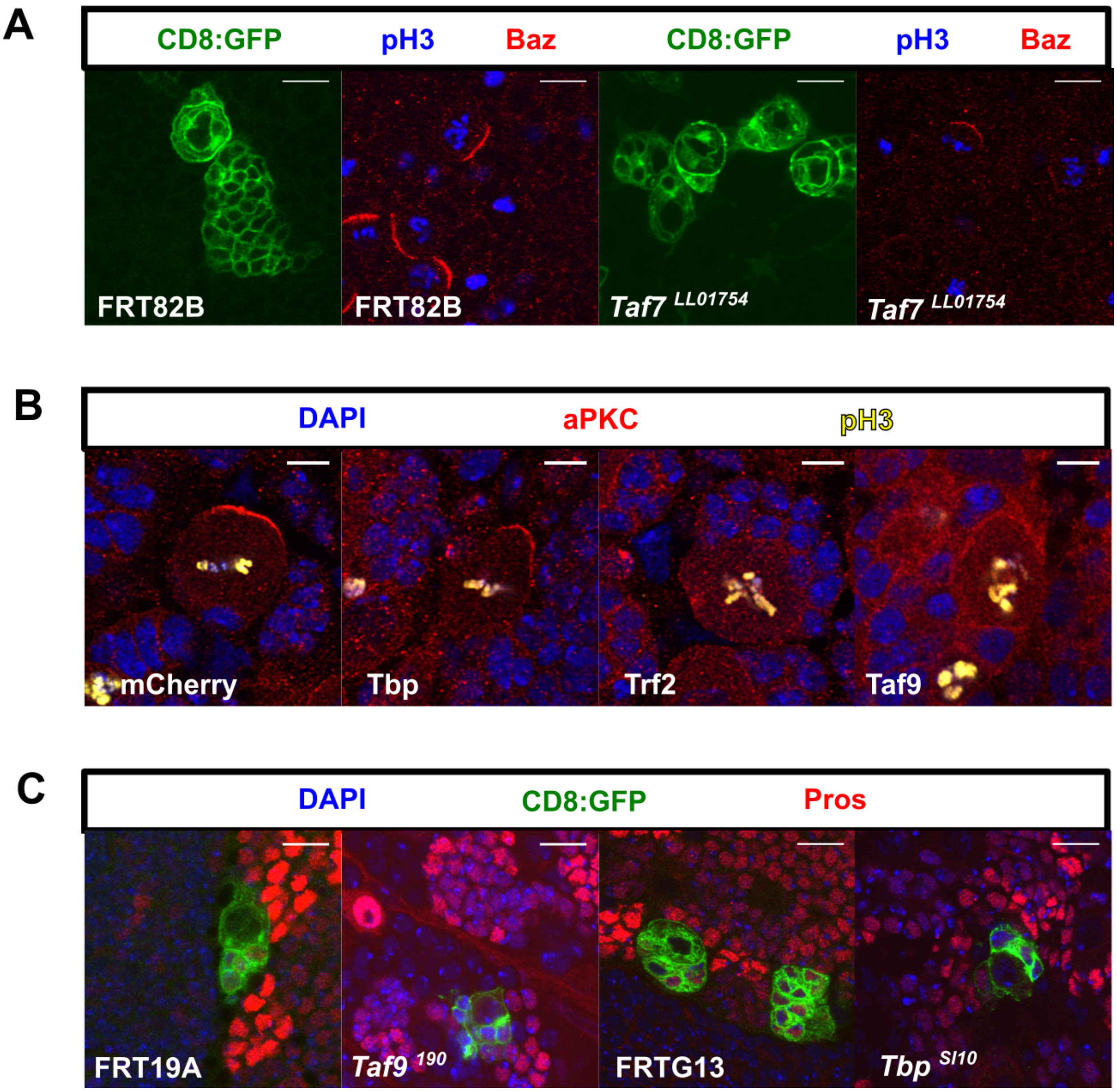

Biology Open • Supplementary information

Figure S4. Analysis of NSC cell polarity and differentiation markers. (A) The NSC polarity marker Baz exhibits defective localization in a subset of *Taf7*^{LL01754} mutant NSCs. Control (FRT82B) or mutant (*Taf7*^{LL01754}) clones are marked with a membrane-tethered GFP, CD8:GFP. (B) The NSC polarity marker aPKC exhibits defective localization in a subset of NSCs depleted for TBP, TRF2 and TAF9. (C) *Taf9*^{190} or *Tbp*^{SI10} mutant NSCs do not accumulate Prospero in the nucleus. Control (FRT19A or FRTG13) or mutant (*Taf9*^{190} or *Tbp*^{SI10}) clones are marked with a membrane-tethered GFP, CD8:GFP. Scale bars represent 20 μm in A and 10 μm in B-D.

Biology Open • Supplementary information

Figure S5

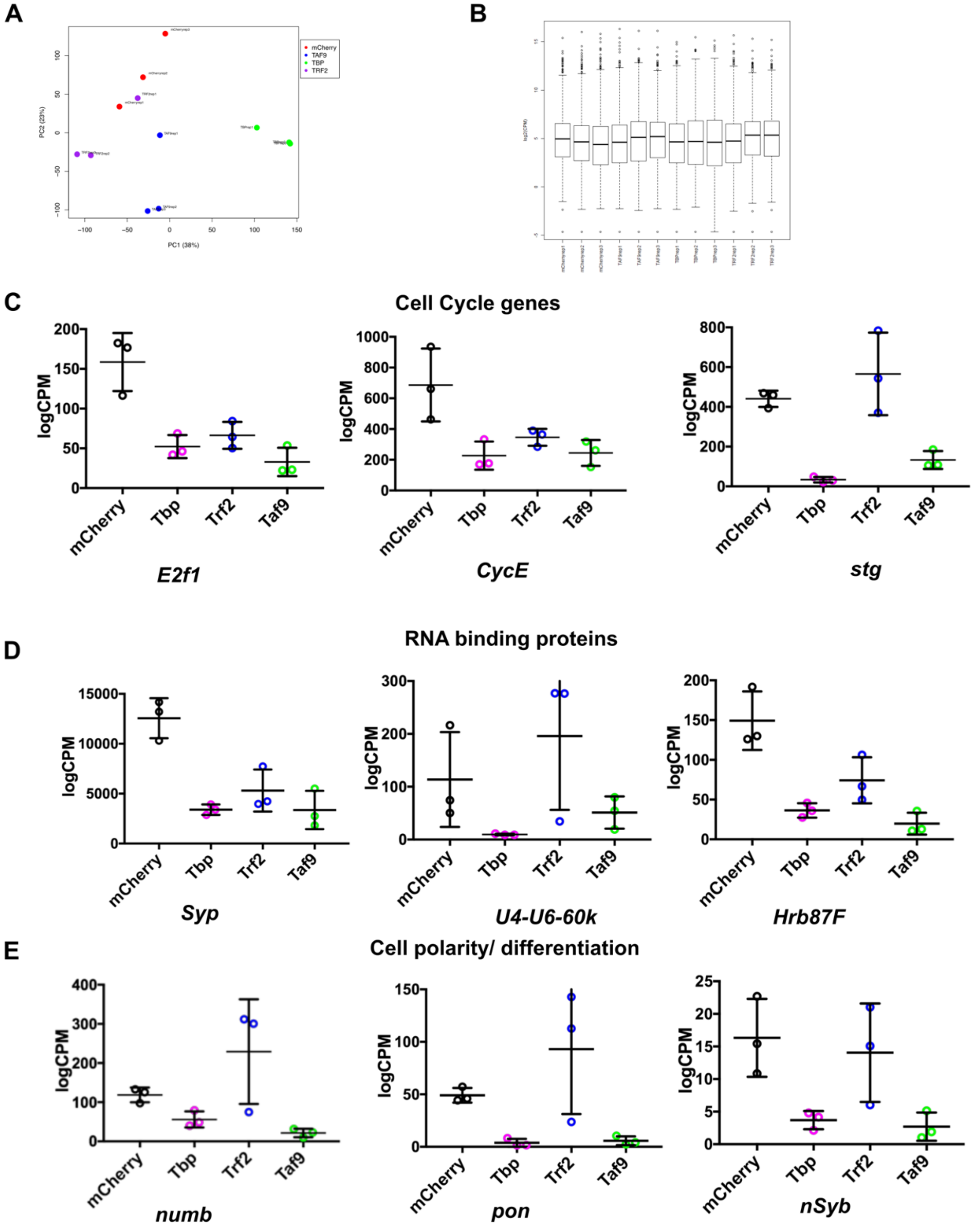

Biology Open • Supplementary information

Figure S5. RNA-seq analysis of FACS-purified NSCs. (A) Principal component analysis of three replicates for each genotype. (B) Box plot depicting normalized reads for three replicates for each genotype. (C) Normalized reads for selected cell cycle genes (*E2f1*, *CycE*, *stg*) in FACS- purified NSCs expressing either control (mCherry), TBP, TRF2 or TAF9 RNAi transgenes. (D) Normalized reads for selected genes encoding RNA binding proteins (*Syp*, *U4-U6-60k*, *Hrb87F*) in FACS- purified NSCs expressing either control (mCherry), TBP, TRF2 or TAF9 RNAi transgenes. (E) Normalized reads for selected differentiation-associated genes (*numb, pon, nSyb)* in FACS- purified NSCs expressing either control (mCherry), TBP, TRF2 or TAF9 RNAi transgenes.

Figure S6

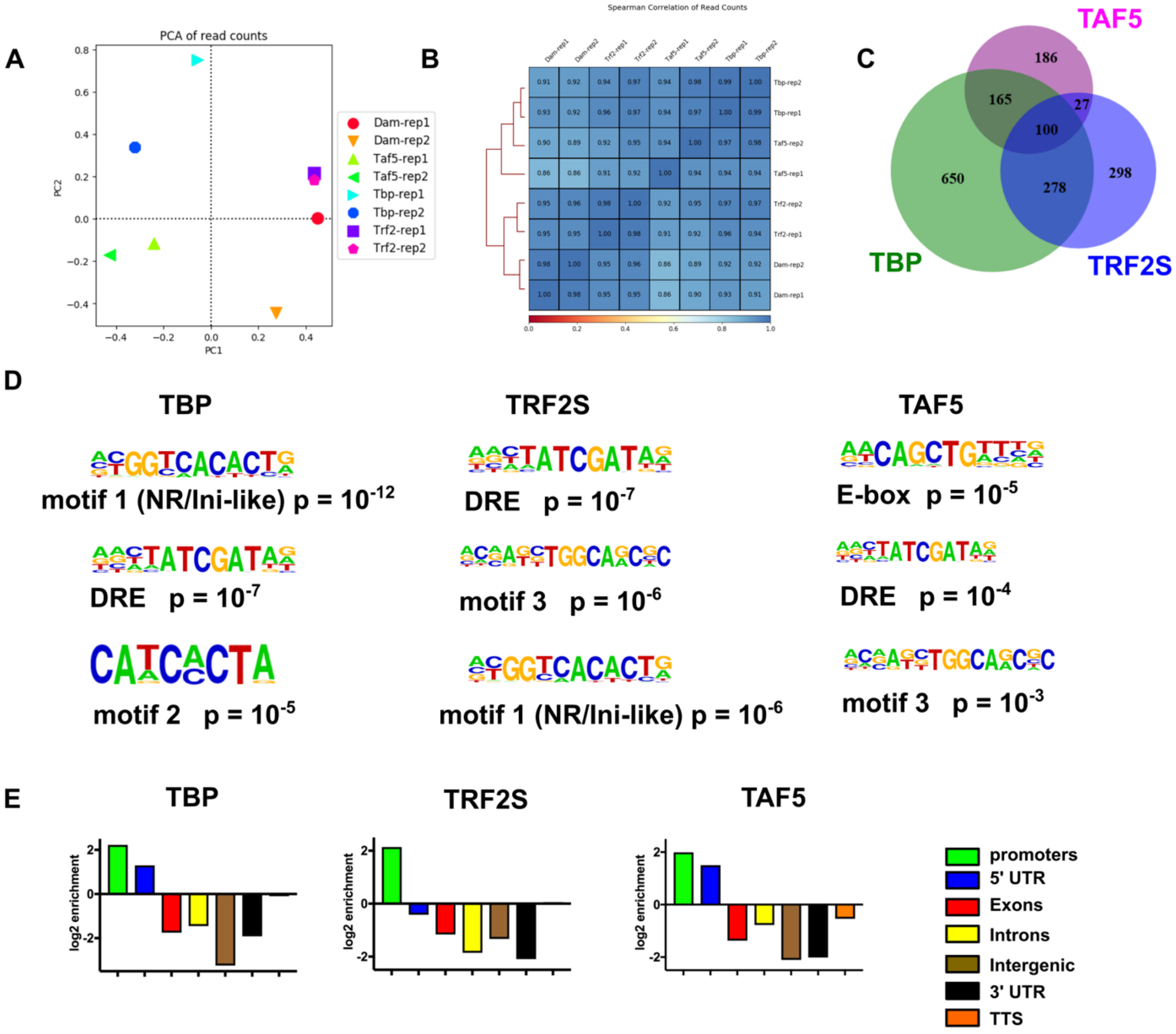

Biology Open • Supplementary information

Figure S6. DamID-seq analysis for Dam-TBP, Dam-TRF2S and Dam-TAF5. (A) Principal component analysis and (B) Spearman correlation of read counts for each of the two replicates for the four Dam proteins (Dam alone, Dam-TBP, Dam-TRF2S, Dam-TAF5). (C) Venn diagram showing the statistically significant overlap between genes with TSS-associated peaks that were common to both replicates. (D) Motif enrichment analysis using HOMER of peaks that were common to both replicates. (E) Genomic distribution of regions bound by Dam-TBP, Dam-TRF2S and Dam-TAF5 in both replicates shows enriched binding at promoters and 5'UTRs. Plots show log2 enrichment scores (observed/expected).

Table S1. List of RNAi lines used and phenotype description.

Click here to Download Table S1

Table S2. List of NSC-expressed genes and identification of differentially expressed genes upon TBP knockdown.

Click here to Download Table S2

Table S3. List of NSC-expressed genes and identification of differentially expressed genes upon TRF2 knockdown.

Click here to Download Table S3

Table S4. List of NSC-expressed genes and identification of differentially expressed genes upon TAF9 knockdown.

Click here to Download Table S4

Table S5. Gene ontology analysis of genes downregulated upon TBP knockdown.

Click here to Download Table S5

Table S6. Gene ontology analysis of genes downregulated upon TAF9 knockdown.

Click here to Download Table S6

Biology Open • Supplementary information
